# Environmental Influences on Stem Cell Behavior

**DOI:** 10.1155/2018/7415460

**Published:** 2018-12-10

**Authors:** Margherita Maioli, Heinz Redl, Martin J. Stoddart

**Affiliations:** ^1^Department of Biomedical Sciences, University of Sassari, Viale San Pietro 43/B, 07100 Sassari, Italy; ^2^Laboratory of Molecular Biology and Stem Cell Engineering, National Institute of Biostructures and Biosystems-Eldor Lab, Innovation Accelerator, CNR, Via Piero Gobetti 101, 40129 Bologna, Italy; ^3^Center for Developmental Biology and Reprogramming (CEDEBIOR), Department of Biomedical Sciences, University of Sassari, Viale San Pietro 43/B, 07100 Sassari, Italy; ^4^Istituto di Ricerca Genetica e Biomedica, Consiglio Nazionale delle Ricerche (CNR), Monserrato, Cagliari, Italy; ^5^Ludwig Boltzmann Institute for Experimental and Clinical Traumatology, AUVA Research Centre, Vienna, Austria; ^6^AO Research Institute Davos, Davos, Switzerland; ^7^Department of Orthopedics and Trauma Surgery, Medical Center Albert-Ludwigs-University Freiburg, Freiburg im Breisgau, Germany

Stem cells are unique elements capable of acquiring a specific phenotype under the exposure of specific stimuli. Within this context, regenerative medicine represents a novel branch of Medicine mainly focused on stem cell-based cellular therapies. For years, scientists developed different kinds of molecules in the attempt to convince stem cells to assume a specific phenotypic identity. These compounds are both natural molecules, for example, melatonin or vitamin D [1, 2], or mixtures of physiological molecules able to act as epigenetic regulators of stem cell fate [3, 4]. This special issue brings together 5 papers to highlight recent developments in the field. Within this landscape, the review by L. Huang and G. Wang perfectly summarizes the effects of different factors used in the past years to orchestrate neural stem cell proliferation and differentiation. This manuscript represents a source for future translational applications of nerve tissue engineering in regeneration after CNS injury. Extracellular vesicles (EVs) are emerging as novel actors for intercellular communication and as a potential diagnostic tool in human diseases. Different cells under physiological and pathological conditions, including tumor cells, produce EVs. The review by I. Laurenzana et al. describes the involvement of EVs in bone marrow-derived stem cell communication, also underlying their role during hematological malignancies as a part of the communication among the niche, HSCs, and MSCs. Recently, studies described the use of physical stimuli in vivo, to induce the patient's own regenerative capabilities, based on stem cell recruitment and fate modulation. Enhanced endogenous response based on posttreatment rehabilitation is driving a new area of regenerative rehabilitation [5–7]. The potential to enhance regenerative processes using physical energies is supported by the ability of electromagnetic fields and mechanical vibrations to drive an efficient reprogramming of the differentiation and regenerative potential of our endogenous stem cells [6–8]. The manuscript by F. Facchin et al. perfectly summarizes these findings, by describing the effect of different kinds of electromagnetic fields and of sound vibrations on stem cell proliferation and differentiation. Stem cell behavior can also be influenced by oxygen concentrations. Upon stimulation, stem cells migrate to more oxygenated areas to heal damaged tissue. Adult stem cells remain in a state of quiescence in their specialized niche until external signals induce a metabolic shift towards an oxidative metabolism [9]. As reported in the research article by A. Menon et al., hypoxia, through the activation of a specific factor, plays a crucial role in preserving stem cells in an undifferentiated state within tissue “hypoxic niches.” The research article by A. Banerjee et al. perfectly fits with this finding, viewing that mesenchymal stromal cells (hAMSCs) from the amniotic membrane can be influenced in their differentiation behavior by high oxygen tension (20%), a condition able also to activate mitochondrial function and induce the production of IL6. We hope that this special issue will introduce novel concepts in understanding stem cell behavior, not only by defining a wide panel of chemical but also physical players in regenerative medicine.
